# Association of IRGM Polymorphisms and Susceptibility to Pulmonary Tuberculosis in Zahedan, Southeast Iran

**DOI:** 10.1100/2012/950801

**Published:** 2012-09-23

**Authors:** Gholamreza Bahari, Mohammad Hashemi, Mohsen Taheri, Mohammad Naderi, Ebrahim Eskandari-Nasab, Mahdi Atabaki

**Affiliations:** ^1^Department of Clinical Biochemistry, School of Medicine, Zahedan University of Medical Sciences, Zahedan 98167-43463, Iran; ^2^Cellular and Molecular Research Center, Zahedan University of Medical Sciences, Zahedan 98167-43463, Iran; ^3^Genetic of Non-Communicable Disease Research Center, Zahedan University of Medical Science, Zahedan 98167-43463, Iran; ^4^Research Center for Infectious Diseases and Tropical Medicine, Zahedan University of Medical Sciences, Zahedan 98167-43463, Iran

## Abstract

Tuberculosis (TB) is a major cause of morbidity and mortality worldwide. IRGM1 is an important protein in the innate immune response against intracellular pathogens by regulating autophagy. Polymorphisms in the IRGM genes are known to influence expression levels and may be associated with outcome of infections. This case-control study was done on 150 patients with PTB and 150 healthy subjects to determine whether the IRGM polymorphisms at positions −1208 A/G (rs4958842), −1161 C/T (rs4958843), and −947 C/T (rs4958846) were associated with PTB. The polymorphisms were determined using tetra-amplification refractory mutation system-PCR (T-ARMS-PCR). The results showed that the IRGM −1161 C/T and −947 C/T polymorphisms were associated with decreased susceptibility to PTB (OR = 0.06, 95% CI = 0.03–0.13, *P* < 0.001 and OR = 0.27; 95% CI = 0.013–0.55, *P* < 0.001, resp.). No significant difference was found among the groups regarding −1208 A/G polymorphism. In conclusion we found that the IRGM −1161 C/T and −947 C/T polymorphisms but not −1208 A/G polymorphism provide relative protection against PTB in a sample of Iranian population.

## 1. Introduction

Tuberculosis is a public health problem especially in developing countries and cause of morbidity and mortality throughout the world. According to the World Health Organization (WHO), it is a global emergency [1] and approximately 2 million peoples annually die due to tuberculosis [2]. Among the one-third of the world infected by TB only 10% develop clinical disease [3]. Increasing evidence indicates that host genetic factors play an important role in susceptibility to TB [3].

Mycobacterium tuberculosis is an intracellular pathogen that can persist within host macrophages. It can reside within phagosomes of macrophages and is able to arrest phagosome maturation [4]. Autophagy is a process in which intracellular components degrade in lysosomes of the cell. It plays a key role against intracellular pathogens such as mycobacterium [5]. It has been shown that immunity-related GTPase (*IRGM*) induced autophagy in macrophages to control M. tuberculosis [6]. This protein is necessary for immunity against a series of intracellular pathogens in mice, including *Listeria*, *Toxoplasma*, and *Mycobacterium tuberculosis* [2]. There are 3 IRG genes, IRGC, IRGQ, and IRGM, in human genome; only IRGM is functional [7].

The IRGM gene with 5 exons is located on chromosome 5q33.1. The first exon is long and encodes 181 amino acids; the four shorter exons extend more than 50 kb downstream from the first exon [8]. Variations in promoter region of IRGM gene have shown to be associated with an increased risk of TB [2, 6].

There is little and controversial data concerning the impact of IRGM polymorphisms and susceptibility to PTB. Therefore, the present study aimed to evaluate the possible association between −1208 A/G, −1161 C/T, and −947 C/T IRGM polymorphisms and pulmonary tuberculosis in a sample of Iranian population. 

## 2. Material and Methods

This case-control study was performed from December 2010 to January 2012 in the Research Center for Infectious Diseases and Tropical Medicine, the Bou-Ali Hospital, Zahedan, Iran. A total of 150 PTB patients and 150 healthy subjects were enrolled in the study. Ethics committee of the Zahedan University of Medical Sciences approved the project and informed consent was taken from all patients and healthy subjects. All control subjects were from the same geographical origin and were living in the same region as the patients with PTB. The diagnosis of PTB was based on clinical, radiological, sputum acid-fast bacillus (AFB) smear positivity, culture, and response to antituberculosis chemotherapy as described previously [9, 10]. Two mL of venous blood was drawn from each subject and genomic DNA was extracted from peripheral blood as described previously [11] and stored at −20°C.

Tetra-primer amplification refractory mutation system polymerase chain reaction (T-ARMS-PCR) is a simple and rapid method for detection of single nucleotide polymorphism (SNP) [12–14]. We designed T-ARMS-PCR for detection of −1208 A/G (rs4958842), −1161 C/T (rs4958843), and −947 C/T (rs4958846) polymorphisms of IRGM gene. We used two external primers (forward outer and reverse outer) and two inner primers (forward inner and reverse inner) for each position that are shown in Table 1.

Polymerase chain reaction (PCR) was done using commercially available PCR premix (AccuPower PCR PreMix, BIONEER, Daejeon, Republic of Korea) according to the manufacturer protocol. Into a 0.2 mL PCR tube containing the AccuPower PCR PreMix, 1 *μ*L template DNA (~100 ng/*μ*L), 1 *μ*L of each primer (10 *μ*M), and 15 *μ*L DNase-free water were added. The PCR cycling conditions were 5 min at 95°C followed by 30 cycles of 30 s at 95°C, 30 s at 63°C for −1208 A/G, 65°C for −1161 C/T, 63°C for −947 C/T, respectively, and 30 s at 72°C, with a final step at 72°C for 10 min to allow for complete extension of all PCR fragments. The PCR products were analyzed by electrophoresis on a 2% agarose gel containing 0.5 *μ*g/mL ethidium bromide and visualized by ultraviolet transilluminator. To ensure genotyping quality, we regenotyped all polymorphisms in random samples and found no genotyping mistake.

**Figure 1:**
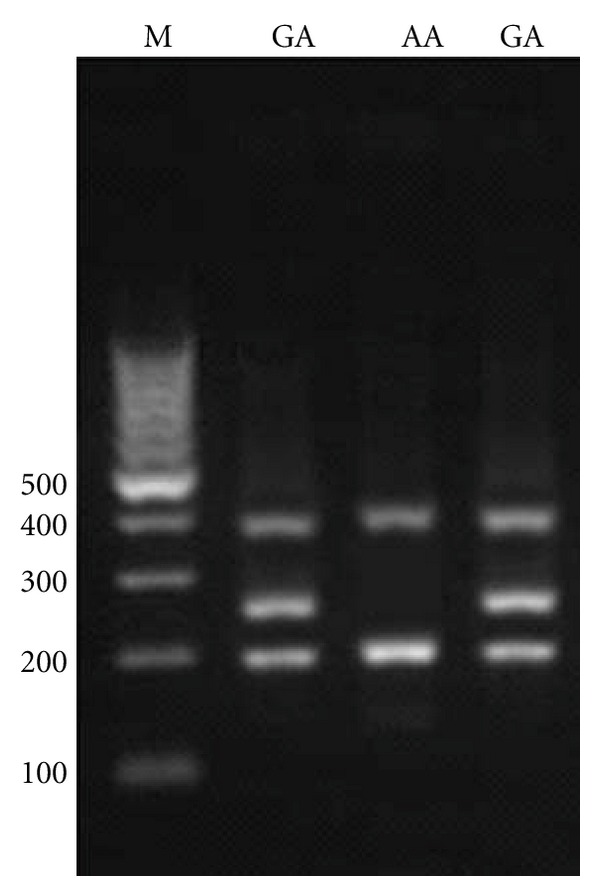
Electrophoresis pattern of tetra-amplification refractory mutation system-polymerase chain reaction (T-ARMS-PCR) for detection of SNP in IRGM −1208 A/G. M : DNA marker. Product sizes were 195 bp for A allele, 254 bp for G allele, and 402 bp for two outer primers (control band).

**Figure 2:**
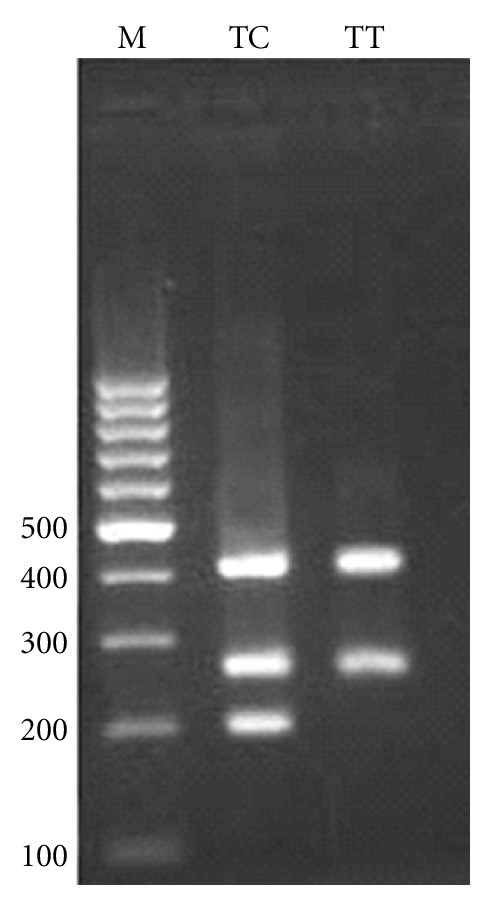
Electrophoresis pattern of tetra-amplification refractory mutation system-polymerase chain reaction (T-ARMS-PCR) for detection of SNP in IRGM −1161 C/T. M : DNA marker. Product sizes were 199 bp for C allele, 261 bp for T allele, and 415 bp for control band.

**Figure 3:**
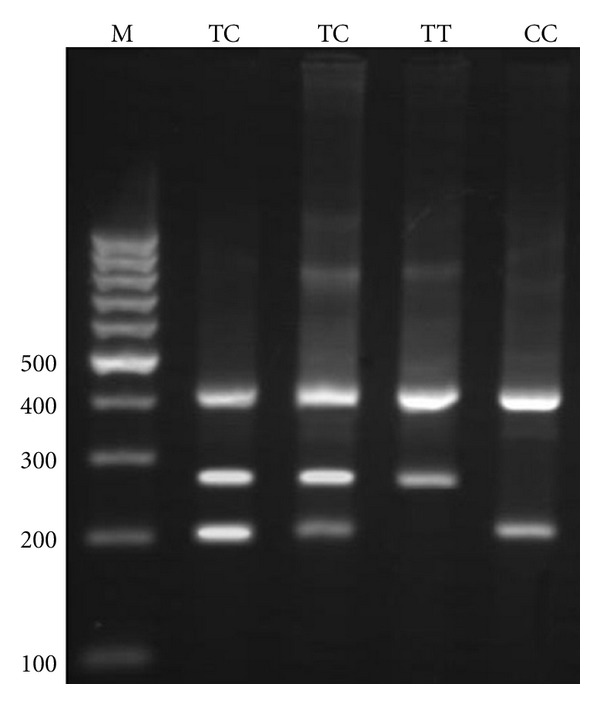
Electrophoresis pattern of tetra-amplification refractory mutation system-polymerase chain reaction (tetra ARMS-PCR) for detection of SNP in IRGM −947 C/T. M : DNA marker. Product sizes were 201 bp for C allele, 263 bp for T allele, and 417 bp for control band.

For −1208 A/G, the PCR product sizes were 195 bp for A allele, 245 bp for G allele, and 402 bp for two outer primers (control band) (Figure 1). For −1161 C/T, product sizes were 199 bp for C allele, 261 bp for T allele, and 415 bp for control band (Figure 2). Product sizes were 201 bp for C allele, 263 bp for T allele, and 417 bp for control band for −947 C/T (Figure 3).

### 2.1. Statistical Analysis

The statistical analysis of the data was performed using the SPSS 18.0 software. Demographics and biochemical parameters between the groups were analyses by independent sample *t*-test for continuous data and *χ* ^2^ test for categorical data. A *P* value less than 0.05 was considered statistically significant. The associations between genotypes of IRGM gene and PTB were estimated by computing the odds ratio (OR) and 95% confidence intervals (95% CI) from logistic regression analyses.

## 3. Results

For determining of IRGM polymorphisms in PTB patients and comparison of these polymorphisms with healthy individual, a total of 150 pulmonary tuberculosis patients with an average age of 47.5 years (59 male, 91 female; minimum 12 years, maximum 78 years) and 150 healthy subjects with a mean age of 44.13 years (53 male, 97 female; minimum 20, maximum 82) were enrolled in the study. There was no significant difference among the groups regarding sex and age (*P* > 0.05). Allele and genotype frequencies of IRGM −1208 A/G, −1161 C/T, and −947 C/T are given in Table 2. No significant difference was found between the groups concerning −1208 A/G polymorphism (*χ* ^2^ = 1.19, *P* = 0.274). A significant difference was observed among case and control groups regarding IRGM −1161 C/T (*χ* ^2^ = 75.37, *P* < 0.001) and −947 C/T (*χ* ^2^ = 15.75, *P* < 0.001). As shown in Table 2, the −1161 CT genotype as well as −1161 C allele was associated with protection from PTB (OR = 0.06, 95%  CI = 0.03–0.13, *P* < 0.001 and OR = 0.36, 95%  CI = 0.26–0.51, *P* < 0.001, resp.). The −947 CT genotype was significantly higher in control group (%23.33) than that in PTB (%8.00) and this polymorphism was negatively associated with susceptibility to PTB (OR = 0.28, 95%  CI = 0.14–0.57, *P* = 0.002). The −947 C allele confers a protective role against PTB (OR = 0.27, 95%  CI = 0.14–0.54, *P* < 0.001).

## 4. Discussion

IRGM1 is an important protein in the innate immune system against TB by regulating autophagy in response to intracellular pathogens. In the present study, we examined the impact of IRGM −1208 A/G, −1161 C/T, and −947 C/T polymorphisms on pulmonary tuberculosis (PTB) risk in a sample of Iranian population. Our finding revealed the protective role of −1161 C/T and −947 C/T polymorphisms against PTB in our population. No significant difference was found between control and PTB groups regarding IRGM −1208 A/G polymorphism. 

To the best of our knowledge, there is little information regarding the association of IRGM polymorphisms and tuberculosis and this is the first report from Iran. The earliest report about the role of IRGM in autophagy and defense against intracellular pathogens is attributed to Singh et al. study [15]. They found that the murine Irgm1 guanosine triphosphatase induced autophagy and generate large autolysosomal organelles for the elimination of intracellular *Mycobacterium tuberculosis*. They also reported that the human IRGM plays a role in autophagy and in the reduction of intracellular bacillary load [15]. 

Intemann et al. for the first time investigated the association between IRGM genotypes and tuberculosis. They found that the IRGM genotype −261 TT provides relative protection against Mycobacterium tuberculosis but not by *M. africanum or M. bovis* [8]. They predicted that −261 T IRGM variant disrupted several transcription factor-binding sitesed and significantly increased expression of the −261 T IRGM variant compared with the −261 C IRGM variant, suggested that TT genotypeing might enhance expression of IRGM protein. They proposed that IRGM and autophagy have a role in protection against *M. tuberculosis* [8]. Che et al. in a study group of Chinese TB patients sequenced 1.7 kb of IRGM promoter region and identified 29 polymorphisms including 11 novel sites [7]. In contrast to our finding they showed that −1208 A allele and −1208 AA genotype of IRGM were associated with decreased susceptibility to TB [7].

A study conducted by King et al. on 370 African American and 177 Caucasian tuberculosis (TB) cases and 180 African American and 110 Caucasian controls showed that single nucleotide polymorphism rs10065172 C/T in IRGM is associated with human vulnerability to TB disease among African Americans [2]. Their results showed that there were not differences in IRGM1 expression level based on genotype. 

A combination of both innate and adaptive immune responses were involved in the host defense against mycobacterium. Autophagy mediates innate immune responses against mycobacterium by promoting phagolysosomal maturation within macrophages [16], besides, autophagy plays a key role in antigen processing and presentation [5]. IRGM involved in the induction of autophagy in macrophages that infected with mycobacterium. Investigation showed that variations of IRGM gene are associated with an increased risk of several diseases such as Crohn's disease and tuberculosis. 

The exact reason as to why only a number of the subjects infected with M. tuberculosis develop clinical disease is clearly unknown. There are some evidences recommending that host genetic factors may be important risk factor for development of tuberculosis [17–20]. The impact of IRGM polymorphism on PTB susceptibility is probably influenced by ethnic background.

In conclusion, our results showed that −1161 C/T and −947 C/T IRGM polymorphisms but not −1208 A/G polymorphism contributes to decreased susceptibility to PTB among an Iranian population. Larger studies with different ethnicitie are required to validate our findings.

## Figures and Tables

**Table 1:** Primers used for polymorphism determination.

Primers	−1208 A/G (rs4958842)	−1161 C/T (rs4958843)	−947 C/T (rs4958846)
Forward outer	TGTGAGTATGTGTGGGCCTGTGCACAGA	GGCATGGGTGAGTGTGCACACC	TCCTCAGCCTTGGCGCCCACTCTA

Reverse outer	AGTTGCTGCCCGTGCCTCTCCCTC	CTAAGCCCCTCACTGCCAGGGG	GCTCATAGGGGAGGCTCGGGCTGT

Forward inner	ACAGCATGCTGGCAGCCCTCGAAA	CAGCCTTGGCGCCCACTCTCGT	CAGAGCAGCCATCCGGCCCCTAC

Reverse inner	AGGCTCCGAGAGCCAGCGAGTGC	GCTGAAGGGCTCCTCAAGTGACG	TAAGCCCCTCACTGCCAGGGGACA

**Table 2:** The genotypes and allele distribution of IRGM polymorphisms in case and control groups.

Polymorphism	PTB *n* (%)	Control *n* (%)	OR (95%CI)	*P*	*OR (95%CI)	*P*
−1208 A/G (rs4958842)						
AA	20 (13.3)	14 (9.3)	Ref.			
AG	130 (86.7)	136 (90.7)	1.49 (0.73–3.08)	0.277	1.53 (0.74–3.18)	0.250
GG	0 (0.0)	0 (0.0)	—	—	—	—
Alleles						
A	170 (56.7)	164 (54.7)				
G	130 (43.3)	136 (45.3)	0.92 (0.67–1.27)	0.681		
−1161 C/T (rs4958843)						
TT	77 (51.3)	9 (6.0)	Ref.			
CT	73 (48.7)	141 (94.0)	0.06 (0.03–0.12)	<0.001	0.06 (0.03–0.13)	<0.001
CC	0 (0.0)	0 (0.0)	—	—	—	—
Alleles						
T	227 (75.7)	159 (53.0)				
C	73 (24.3)	141 (47.0)	0.36 (0.26–0.51)	<0.001		
−947 C/T (rs4958846)						
TT	138 (92.0)	113 (75.3)	Ref.			
CT	12 (8.0)	35 (23.4)	0.28 (0.14–0.57)	0.002	0.27 (0.13–0.55)	<0.001
CC	0 (0.0)	2 (1.3)	—	—	—	—
CT + CC	12	37 (24.66)	0.26 (0.13–0.53)	<0.001	0.26 (0.13–0.53)	<0.001
Alleles						
T	288 (96.0)	261 (87.0)				
C	12 (4.0)	39 (13.0)	0.27 (0.14–0.54)	<0.001		

*Adjusted for age and sex.
